# Mutagenic consequences of cytosine alterations site-specifically embedded in the human genome

**DOI:** 10.1186/s41021-016-0045-9

**Published:** 2016-09-01

**Authors:** Akira Sassa, Yuki Kanemaru, Nagisa Kamoshita, Masamitsu Honma, Manabu Yasui

**Affiliations:** 1 Division of Genetics and Mutagenesis, National Institute of Health Sciences, 1-18-1 Kamiyoga, Setagaya-ku, Tokyo, 158-8501 Japan; 2 Present address: Division of Toxicology, Department of Pharmacology, Toxicology and Therapeutics, Showa University School of Pharmacy, Tokyo, 142-8555 Japan

**Keywords:** Mutagenesis, Gene targeting, Deamination, Mutagenic potential

## Abstract

**Introduction:**

Cytosine residues in CpG dinucleotides often undergo various types of modification, such as methylation, deamination, and halogenation. These types of modifications can be pro-mutagenic and can contribute to the formation of mutational hotspots in cells. To analyze mutations induced by DNA modifications in the human genome, we recently developed a system for tracing DNA adducts in targeted mutagenesis (TATAM). In this system, a modified/damaged base is site-specifically introduced into intron 4 of thymidine kinase genes in human lymphoblastoid cells. To further the understanding of the mutagenesis of cytosine modification, we directly introduced different types of altered cytosine residues into the genome and investigated their genomic consequences using the TATAM system.

**Findings:**

In the genome, the pairing of thymine and 5-bromouracil with guanine, resulting from the deamination of 5-methylcytosine and 5-bromocytosine, respectively, was highly pro-mutagenic compared with the pairing of uracil with guanine, resulting from the deamination of cytosine residues.

**Conclusions:**

The deamination of 5-methylcytosine and 5-bromocytosine rather than that of normal cytosine dramatically enhances the mutagenic potential in the human genome.

## Introduction

**Fig. 1:**
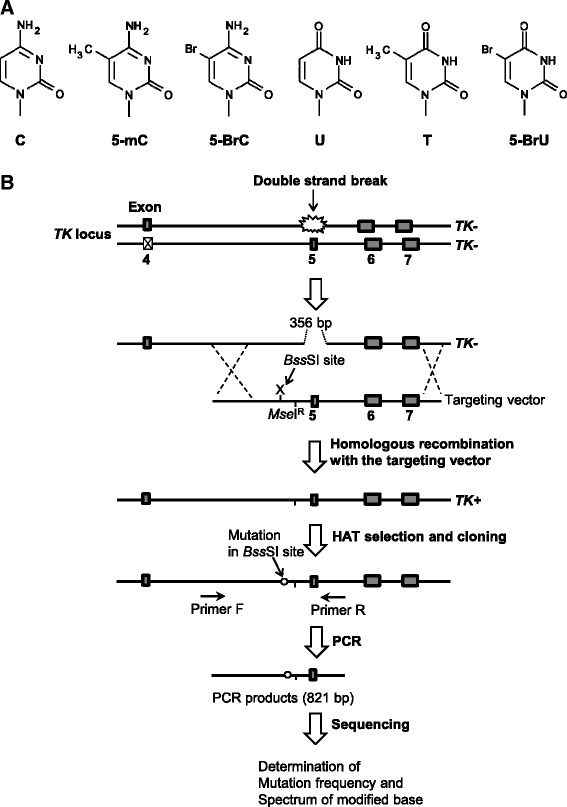
Overview of the TATAM system. Structures of cytosine alteration (**a**) and the principle of the TATAM system (**b**). X on the targeting vector indicates the position of cytosine, 5-mC, 5-BrC, U, 5-BrU, or thymine at the *Bss*SI site. The targeting vectors pvINT^C:G^, pvINT^5mC:G^, pvINT^5BrC:G^, pvINT^U:G^, pvINT^5BrU:G^, or pvINT^T:G^ and the I-*Sce*I expression plasmid pCBASce were co-transfected into TSCER122 cells. Double-strand break at the I-*Sce*I site enabled gene targeting by inducing site-specific homologous recombination. The targeting vector contained an *Mse*I^R^ site that was resistant to *Mse*I digestion and thereby distinguished targeted and non-targeted revertants of *TK. TK* revertants were selected by using HAT. Genomic DNA of the revertant colonies was prepared, and part of the *TK* gene containing the modified DNA integrated site was amplified by PCR. The amplified fragment was sequenced as described in the Materials and Methods section

CpG dinucleotides in the genome are subjected to various types of modification including cytosine methylation. The methylation of cytosine to 5-methylcytosine (5-mC) is a common DNA modification and is important for the epigenetic mechanism of gene regulation in higher eukaryotes. In mammalian cells, 3–6 % of cytosine residues and 70–80 % of cytosine residues in CpG dinucleotides are methylated [1–3]. Such cytosine residues often undergo inappropriate modifications (Fig. 1a), leading to genomic instability.

Cytosine and 5-mC in the genome are often spontaneously deaminated to form U:G and T:G mismatches, respectively [4]. These mismatches are also produced by enzymatic deamination caused by activation-induced deaminase or apolipoprotein B mRNA editing enzyme, catalytic polypeptide-like 3A (APOBEC3A) [5–7]. The resultant uracil and thymine can pair with adenine during DNA replication, causing C:G to T:A transition mutations. In fact, cytosine residues at CpG dinucleotides in the tumor suppressor gene *TP53* is known as a mutational hotspot in carcinoma cells [8]. It has been suggested that in DNA, the hydrolytic deamination of 5-mC occurs more rapidly than that of cytosine [9, 10].

Cytosine modification also occurs during chronic inflammation. At inflammation sites, phagocytic cells generate peroxidases that produce reactive oxidants such as hypobromous acid and hypochlorous acid [11–13]. These oxidants can result in several types of halogenated DNA damages, leading to mutagenesis [14–20]. Among them, the halogenation of cytosine is detrimental to organisms. For example, 5-bromocytosine (5-BrC) and 5-chlorocytosine (5-ClC) in DNA can potentially compromise epigenetic signals by mimicking 5-mC [21]. Moreover, 5-BrC is converted to 5-bromouracil (5-BrU) by APOBEC3A [22], which may result in enhanced mutagenesis in the genome.

The mutagenesis of modified/deaminated cytosine residues has been extensively studied in *Escherichia coli* and in plasmids introduced into mammalian cells [4, 10, 23–27]. However, the mutagenic consequences of such alterations in the human genome are yet to be completely understood. We recently developed a system for tracing DNA adducts in targeted mutagenesis (TATAM) by directly introducing a DNA modification site specifically into intron 4 of the thymidine kinase (*TK*) gene in human lymphoblastoid cells (Fig. 1b) [28]. In this study, for better understanding the mutagenesis of cytosine modification *in vivo*, we introduced cytosine, 5-mC, and 5-BrC paired with guanine and their deamination products U:G, T:G, and 5-BrU:G mismatch at CpG dinucleotides in the genome using the TATAM system.

## Materials and methods

### Cell culture

The human lymphoblastoid TSCER122 cells used were derivatives of TK6 cells, as reported previously [28]. Cells were maintained in RPMI 1640 medium (Nacalai Tesque) supplemented with 10 % horse serum (JRH Biosciences), 200 μg/ml of sodium pyruvate, 100 U/ml of penicillin, and 100 μg/ml of streptomycin at 37 °C under 5 % CO_2_ and 100 % humidity.

### Outline of the TATAM system

TSCER122 cells are compound heterozygous for the *TK* gene (*TK* −/−) because of the complete loss of exon 5 in one allele and a point mutation in exon 4 in the other (Fig. 1b). Because there is an I-*Sce*I recognition site in the original exon 5 region, the expression of the I-*Sce*I enzyme in TSCER122 cells generated a double-strand break in the *TK* gene, allowing for the generation of the wild type *TK* (*TK* +/−) by homologous recombination with the targeting vector. TSCER122 cells were co-transfected with the I-*Sce*I expression plasmid and the targeting vector site-specifically containing a synthetic DNA adduct. After 3 days incubation, cells were seeded 96-well plates in the presence of hypoxanthine, aminopterin, and thymidine (HAT) to isolate the DNA adduct-integrated revertant clones. Subsequently, the *TK* gene loci of the revertant clones were sequenced (Fig. 1b).

### Preparation of site-specific modified targeting vector

**Fig. 2:**
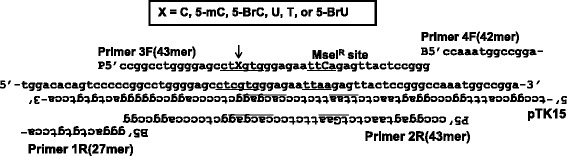
Details of the site of modification. The position of a modification is indicated by X in the primer sequence. An unmodified cytosine, 5-mC, 5-BrC, U, 5-BrU, or thymine paired with guanine was inserted at the *Bss*SI site. The *Mse*I^R^ site was placed near the *Bss*SI site

The targeting vectors pvINT^C:G^, pvINT^5mC:G^, pvINT^5BrC:G^, pvINT^U:G^, pvINT^5BrU:G^, and pvINT^T:G^ containing C:G, 5-mC:G, 5-BrC:G, U:G, 5-BrU:G, and T:G base pairs, respectively, in place of the underlined cytosine/guanine at the *Bss*SI site (5′–CTCGTG/5′–CACGAG) were prepared by a polymerase chain reaction (PCR)-based method with the plasmid pTK15, as previously described (Fig. 2) [28, 29]. A 5′–TTCA sequence (*Mse*I^R^) was labeled near the modified *Bss*SI site. This modified site was resistant to *Mse*I digestion and thus distinguished targeted and non-targeted revertants of *TK* according to an interallelic recombination (Fig. 1b). The vectors were sequenced to confirm the presence of the modified cytosine at the expected site.

### Transfection and cloning of TK revertant cells

DNA transfection was performed as previously described [28]. Briefly, the targeting vector (1 μg) and I-SceI expression plasmid pCBASce (50 μg) were co-transfected into 5 × 10^6^ cells that were suspended in 0.1 ml of Nucleofector Solution V (Lonza) using Nucleofector I, in accordance with the manufacturer’s instructions. After incubation for 72 h, cells were seeded into 96-microwell plates in the presence of HAT (200 μM hypoxanthine, 0.1 μM aminopterin, and 17.5 μM thymidine) for isolating targeting vector-integrated revertant clones. After incubation for 2 weeks, drug-resistant colonies (*TK* revertants) were analyzed.

### Mutation analysis

Genomic DNA templates for PCR were prepared from *TK*-revertant colonies using alkaline lysis, as previously described [30]. Briefly, cells were treated with 18 μl of 50 mM NaOH at 95 °C for 10 min and neutralized by adding 2 μl of 1 M Tris-HCl (pH 8.0). The cell lysates were then used as templates for PCR to amplify the *TK* gene fragments containing the modified cytosine integration site. PCR was performed using KOD FX (Toyobo) with the following primers: forward primer 5′–GCT CTT ACG GAA AAG GAA ACA GG–3′ and reverse primer 5′–CTG ATT CAC AAG CAC TGA AG–3′. The resulting DNA fragments were sequenced using an ABI 3730×l DNA analyzer (Applied Biosystems), and clones harboring the *Mse*I^R^ sequence were counted for determining the frequency of modified cytosine integration and numbers of mutations at the *Bss*SI site. The integration frequency of the modified cytosine was calculated by dividing the number of *Mse*I^R^ clones by the total number of revertant clones analyzed. A single point mutation was defined as a single base substitution, insertion, or deletion detected at the modified cytosine. Multiple mutations were multiple base substitutions, deletions, and/or insertions that were detected at sites including the modified cytosine. Base substitutions, deletions, and/or insertions found at sites other than the modified cytosine were defined as non-targeted. Mutant proportions were calculated by dividing the number of mutants by the number of *Mse*I^R^-bearing clones.

### Statistical analysis

Statistical significance was evaluated by Fisher’s exact test. *P*-values less than 0.01 were considered to be statistically significant.

## Results and discussion

To investigate the mutagenic potential of cytosine alterations in the genome, targeting vectors pvINT^C:G^, pvINT^5mC:G^, pvINT^5BrC:G^, pvINT^U:G^, pvINT^5BrU:G^, and pvINT^T:G^ were prepared, containing C:G, 5-mC:G, 5-BrC:G, U:G, 5-BrU:G, and T:G base pairs, respectively, as previously reported [28]. The revertant frequencies were comparable between the targeting vectors used (data not shown), indicating that the modified residues on the targeting vector did not influence the efficiency of homologous recombination.

### Mutagenic potential of 5-methylcytosine and 5-bromocytosine in the genome

**Fig. 3:**
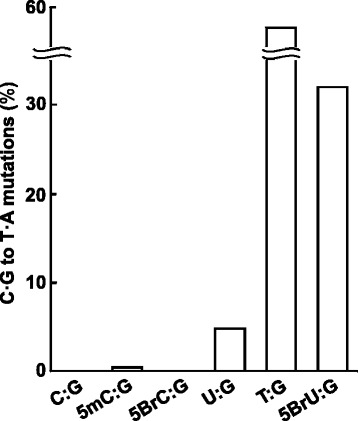
Proportions of C:G to T:A transition mutations induced by the integration of the targeting vector. Proportions of C:G to T:A transition mutations induced by the integration of pvINT^C:G^, pvINT^5mC:G^, pvINT^5BrC:G^, pvINT^U:G^, pvINT^5BrU:G^, and pvINT^T:G^ in TSCER122 cells. Data are derived from at least two independent transfections. The results are also tabulated in Table 1

As shown in Table 1, the total proportion of mutants induced by the integration of pvINT^C:G^, the control vector, was 1.5 %; no C:G to T:A transition mutations were observed (Fig. 3). When pvINT^5mC:G^ was integrated, the proportion of mutants (1.4 %) was comparable to that of pvINT^C:G^. Some C:G to T:A transition mutations (0.44 %) were detected, followed by one base deletion (0.20 %), one base insertion (0.20 %), and non-targeted mutations, referred to as “others” (0.59 %), indicating that 5-mC itself enhances C:G to T:A transition mutations via its deamination, but the frequency is below that of background mutations in this system. This is in agreement with the finding that the frequency of mutations induced by 5-mC ranges from 10^−3^ to 10^−7^ in *E. coli* with different genetic backgrounds [10, 22, 31].

**Table 1:** Mutation spectra induced by integration of the targeting vectors

Targeting vector	TK revertants analyzed	X:G-integrated revertants^a^	No mutation	Single point mutation^b^					Multiple^e^	Others^f^	Total mutation	ND^g^
				T	G	A	Del^c^	Ins^d^				
pvINT^C:G^	457	410 (100 %)	403 (98 %)	0	0	0	2 (0.49 %)	1 (0.24 %)	0	3 (0.73 %)	6 (1.5 %)	1
pvINT^5mC:G^	722	676 (100 %)	667 (99 %)	3 (0.44 %)	0	0	1 (0.20 %)	1 (0.20 %)	0	4 (0.59 %)	9 (1.4 %)	0
pvINT^5BrC:G^	778	705 (100 %)	700 (99 %)	0	2 (0.28 %)	0	0	0	0	3 (0.43 %)	5 (0.71 %)	3
pvINT^U:G^	369	335 (100 %)	309 (92 %)	16 (4.8 %)	2 (0.60 %)	0	1 (0.30 %)	2 (0.60 %)	0	3 (0.90 %)	34 (8.1 %)	2
pvINT^T:G^	187	176 (100 %)	77 (44 %)	98 (56 %)^h^	0	0	0	0	0	0	98 (56 %)	1
pvINT^5BrU:G^	619	524 (100 %)	349 (67 %)	167 (32 %)^h^	4 (0.76 %)	0	1 (0.19 %)	0	2 (0.38 %)	1 (0.19 %)	175 (33 %)	2

^a^X:G indicates C:G, 5-mC:G, 5-BrC:G, U:G, T:G, 5-BrU:G mispair

^b^A single base substitution, one-base insertion, or one-base deletion detected at the modified base

^c^One-base deletion

^d^One-base insertion

^e^Multiple base substitutions, deletions, and/or insertions detected at sites including the modified base in the *Bss*SI site

^f^Mutations found at sites other than the modified base

^g^Not detectable

^h^ *P* < 0.01 (significant difference versus pvINT^U:G^)

Regarding halogenated cytosine, it has been suggested that 5-ClC causes C:G to T:A transition mutations at rates ranging from 5 to 9 % by mispairing with adenine in *E. coli* [32]. Based on our results, however, 5-BrC did not induce C:G to T:A transition mutations (0 %, Fig. 3 and Table 1). The total proportion of mutants induced by pvINT^5BrC:G^ (0.71 %) was comparable to that of the control vector. This low pro-mutagenicity of 5-BrC is consistent with an *in vitro* analysis demonstrating that human DNA polymerases bypass 5-BrC without detectable miscoding [19]. The inconsistency between the previous study on 5-ClC and our results for 5-BrC is probably due to the different atomic radii of the halogens, effects of the specific DNA sequence context, or distinct repair mechanisms between *E. coli* and human cells.

### Mutagenic potential of U:G and 5-BrU:G mismatch in the genome

The integration of pvINT^U:G^ mainly induced C:G to T:A transition mutations (4.8 %), and the total proportion of mutants was 8.1 % (Fig. 3 and Table 1). This mutagenesis caused by the U:G mismatch in the genome is consistent with that in previous reports describing the well-known pro-mutagenicity of the uracil residue [4, 33]. Furthermore, the proportion of mutants was dramatically enhanced when pvINT^5BrU:G^ was integrated (33 %), resulting in an approximately 7-fold higher proportion of C:G to T:A transition mutations than that occurring when pvINT^U:G^ was integrated (4.8 %) (Fisher’s exact test, *P* < 0.01). This indicates that a bromine atom at the 5′–position of uracil interferes with repair using enzymes such as DNA glycosylases in the genome, thereby resulting in enhanced mutagenesis.

### Mutagenic potential of T:G mismatch in the genome

Unexpectedly, the integration of the T:G mismatch (pvINT^T:G^) accounted for the highest proportion of mutants (56 %) (Table 1). Notably, all these mutants harbored C:G to T:A transition mutations, and the proportion of such mutations was 12-fold higher than that associated with the integration of a U:G mismatch (4.8 %) (Fisher’s exact test, *P* < 0.01) (Fig. 3). This high pro-mutagenicity of T:G mispairing is in contrasts with a previous report describing that T:G mismatches in episomal DNA are preferentially repaired to C:G at an approximate efficiency of 90 % by mismatch repair in mammalian cells [27]. Although our cell lines are mismatch repair proficient [34], the integrated T:G mismatch in the *TK* locus did not seem to have been corrected. Therefore, the repair efficiency of the T:G mismatch by the specific mismatch repair might depend on the genomic loci where the mismatch has been integrated. Our *in vivo* results are in agreement with those in a previous *in vitro* study demonstrating that the repair of mismatched T:G is far less efficient than that of mismatched U:G at a mutational hotspot sequence in the *TP53* gene [35].

On the basis of our results, T:G and 5-BrU:G mismatches, resulting from the deamination of 5-mC:G and 5-BrC:G, respectively, markedly enhanced the mutagenic potential compared with that of the U:G mismatch. Although it has been suggested that human thymine DNA glycosylase and methyl-CpG binding protein 4 excise thymine and 5-BrU paired with guanine at CpG dinucleotides [21, 36–38], they might play minor roles in repair in cells. Thus, once deamination of the modified cytosine occurs, the deaminated residues could steadily induce mutations. Because the frequencies of C:G to T:A transition mutations induced by 5-mC and 5-BrC were 0.44 % (4.4 × 10^−3^) and 0 % (<10^−3^), respectively (Table 1), the frequencies of deamination of them might be equal to or less than the order of 10^−3^ in TSCER122 cells. Taking these findings together, we emphasize that those deaminated bases contribute to the mutagenesis and formation of mutational hotspots at specific loci, for example, CpG dinucleotides, in the genome.

## Conclusion

Overall, we revealed the mutagenic potential of modified/deaminated cytosine residues in the human genome. Because T:G and 5-BrU:G mismatches can be highly pro-mutagenic, the rate-limiting step in the formation of mutational hotspots might be the deamination of modified cytosine residues. Our results are also useful to further study the mechanisms by which genomic integrity is maintained.

## Abbreviations

5-BrC: 5-bromocytosine
5-BrU: 5-bromouracil
5-ClC: 5-chlorocytosine
5-mC: 5-methylcytosine
PCR: polymerase chain reaction
TATAM: tracing DNA adducts in targeted mutagenesis
TK: thymidine kinase
